# Boiling points: Profiling individual differences in frustration triggers and affective responses

**DOI:** 10.1371/journal.pone.0353785

**Published:** 2026-08-12

**Authors:** Wasifa R. Orthy, Benjamin Beygi, Hannaneh Yazdi

**Affiliations:** 1 Department of Neuroscience, Amherst College, Amherst, Massachusetts, United States of America; 2 Department of Medicine, Karolinska Institutet, Stockholm, Sweden; 3 Department of Clinical Neuroscience, Karolinska Institutet, Stockholm, Sweden; 4 Department of Physiology and Pharmacology, Karolinska Institutet, Stockholm, Sweden; Universiti Sains Malaysia, MALAYSIA

## Abstract

Frustration is a universally experienced yet understudied affective state, frequently examined in isolated, controlled laboratory environments that neglect the intricate interaction of internal and situational factors. This exploratory research extends beyond such isolated settings by investigating how individual differences, including personality traits, behavioral tendencies, and situational triggers, influence the onset, severity, and reactions to frustration. A total of 145 participants (100 women), aged between 14 and 66 years, completed the Frustration Induction Task (FIT), which was designed to elicit frustration through factors such as time constraints, disruptive notifications, ambiguous feedback, and keyboard manipulation. Based on participants self-reported experiences of frustration, three distinct response profiles were identified: High Frustration, Mid Frustration, and Low Frustration. The High Frustration profile showed descriptively higher neuroticism, emotion-regulation difficulties, and discomfort intolerance, whereas the Low Frustration profile showed descriptively higher emotional intelligence and personal standards of perfectionism. However, individual personality traits and behavioral tendencies did not differ significantly across profiles after correction for multiple comparisons. Situational triggers, particularly time pressure and evaluative feedback more reliably differentiated the frustration profiles. These results provide a more comprehensive understanding of individual differences in frustration responses and emphasize the importance of developing tailored emotion regulation strategies in both clinical settings and high-stress environments.

## Introduction

Frustration arises when effort encounters unexpected blockages, transforming minor setbacks into emotional disruptions [1]. While often blamed on situational triggers, factors such as personality traits and behavioral tendencies may also significantly influence how frustration is experienced [2]. Some may respond with outbursts, others with withdrawal, especially under situational triggers such as time pressure, unpredictability, or cognitive strain [3,4]. Frustration has typically been studied in fragments, researching personality traits, situational triggers, and affective responses separately rather than exploring how they may intertwine. This exploratory study aims to unify these components by examining how personality traits and behavioral tendencies influence frustration triggers, with the goal of better understanding individuals prone to frustration and taking an initial step toward a more comprehensive approach to frustration responses.

Frustration is a familiar, often unwelcome companion in daily life. It is an emotional response that occurs when individuals perceive unexpected barriers to achieving their objectives [5]. In the present study, frustration was operationally defined as a self-reported negative affective state arising when goal-directed task performance was obstructed or disrupted. This definition was examined experimentally using the Frustration Induction Task (FIT).

Frustration lurks behind a slow-loading webpage, a stubborn math problem, or an uncooperative colleague, and it strikes when effort meets resistance, leaving a trail of annoyance, anger, or dejection in its wake. It is often characterized by agitation, dissatisfaction, and, in more severe cases, aggression [1]. While frustration is frequently regarded as a response to external obstacles, it is also influenced by internal perceptions, such as the perceived significance of the goal, the individual’s belief in their ability to control the situation, and the extent of effort already invested [6,7]. In many cases, feeling frustrated can act as a motivator, pushing individuals to persist and problem-solve [8]. However, when repeated failures occur, frustration can escalate into emotional distress, which in turn influences decision-making and social interactions [9]. While the behavioral manifestation of frustration may range from simple annoyance to aggressive outbursts, the underlying psychological processes are often far more complex [10,11].

Consider a shopper in a busy retail setting stuck at checkout due to a malfunctioning payment system. As the queue behind them grows, so does the contextual pressure. Will they stay calm, or will their frustration turn into visible agitation? Their response is shaped by whether they blame external factors, such as faulty technology, or internal ones, like personal errors [11]. In one scenario, they may react with irritation toward the store’s inefficiency, and in another, they might question their own actions, leading to self-directed frustration or embarrassment [12]. A similar pattern emerges in human-computer interactions: when digital systems crash or freeze during critical tasks, frustration can quickly escalate, sometimes leading to task abandonment or aggressive outbursts, such as slamming a keyboard or venting at support staff [13].

Consequently, frustration exhibits considerable variability among individuals, despite being a prevalent emotional experience, which is influenced by personality traits and behavioral tendencies [14]. Individuals exhibiting elevated levels of neuroticism tend to demonstrate reduced tolerance to frustration and exhibit more pronounced emotional reactions [15]. Conversely, personality characteristics such as agreeableness and conscientiousness are associated with increased frustration tolerance, as these traits support the development of more effective coping strategies and emotional regulation mechanisms [7]. Additionally, behavioral tendencies such as high perfectionism, low self-esteem, and impulse control amplify frustration experiences, potentially increasing vulnerability to adverse emotional and behavioral outcomes [16,17]. Individual responses to frustration also differ, as some may externalize their feelings through aggression or emotional outbursts [18], whereas others may internalize frustration, responding by withdrawing or becoming disengaged from challenging tasks [19,20].

Beyond individual predispositions, frustration thrives in environments where external pressure, difficulty, and unpredictability collide [21]. Among the most potent frustration triggers is the combination of time pressure and task difficulty, two factors that, when paired, create a high-stakes environment with little room for mistakes [22]. When individuals perceive that they have little to no control over the situation, frustration intensifies, transforming what might have been a minor inconvenience into a source of significant distress [23]. The more frustration-inducing elements accumulate, such as cognitive overload [5], unexpected changes in tasks [8], and external disruptions like technical malfunctions [7], the more likely an individual is to experience greater emotional dysregulation.

However, these individual differences in experiencing frustration are often investigated separately in research related to the study of frustration, without consideration for how they may interact within the broader psychological landscape. This fragmented approach limits our understanding of frustration as an interconnected process, in which personality traits, behavioral tendencies, and situational triggers may work together to shape the emotional response to frustration arousal. Building on this, this study aims to examine the key frustration triggers and determine their roles in predicting the onset and intensity of frustration. It also evaluates which of the big five personality traits are the strongest moderators of frustration responses under experimentally manipulated conditions. In addition to personality traits, this study investigates behavioral tendencies to explore how they moderate frustration levels during the same task. Finally, the study examines whether distinct experience profiles exist based on how individuals respond to frustration.

By integrating these psychological, situational, and affective components, this exploratory research seeks to uncover the nuanced psychological mechanisms that shape how frustration unfolds across individual differences. These findings can help mental health interventions by enhancing emotional regulation strategies, support systems, and therapeutic approaches across clinical, educational, and workplace landscapes.

## Methods

### Participants

The study used a non-probability convenience sampling strategy. This approach was selected because the study was exploratory and aimed to recruit a heterogeneous participant pool with variability in frustration responses, personality traits, and behavioral tendencies, rather than to estimate population prevalence from a representative sample. The target sample size was determined based on a power analysis for the planned continuous analyses examining associations between frustration responses and individual-difference or task-related variables. Because the number of frustration profiles was not known in advance and was identified empirically through exploratory clustering, the sample size calculation was not based on a prespecified number of groups. Assuming α = .05, 80% power, and a medium expected association of r = .25, the analysis indicated that approximately 123 participants would be required. The initial sample consisted of N = 147 exceeding the target and providing adequate sensitivity for detecting medium-sized associations. Two participants were excluded from the final sample: one because of a technical issue and one because they withdrew before completing the experiment. The final sample consisted of 145 participants, including 100 females (68.97%), with overall ages ranging from 14 to 66 years (*M* = 28.35, *SD* = 9.15). The broad age range was permitted because the study was designed as an exploratory investigation of frustration responses in a heterogeneous participant pool rather than as a developmental or age-stratified comparison. Age was not used as an experimental grouping factor; however, age differences across frustration clusters were examined statistically to evaluate whether cluster membership was associated with age. Participants were eligible for inclusion if they were able to understand the study instructions, provide written informed consent or assent with guardian consent when applicable, and complete the iPad-based Frustration Induction Task. Exclusion criteria were withdrawal before completion of the study or technical difficulties that prevented valid completion of the experimental task. Two participants were excluded according to these criteria: one due to technical difficulties that interrupted task completion, and one due to voluntary withdrawal. The recruitment period of this study took place between 2024-11-21 and 2024-12-19. The study received ethical approval from the Swedish Ethical Review Authority (Dnr 2024-04648-01) and was conducted in full accordance with the Declaration of Helsinki. Prior to participation, all individuals received detailed information about the study procedures and their right to withdraw. Written informed consent was obtained from all adult participants. For participants under the age of 18, written consent was obtained from a parent or legal guardian in addition to the participant’s assent.

### Recruitment

Participants were recruited via the Karolinska Institutet Psychology Testing Recruitment System, online advertisements (www.accindi.se), and community notice boards to establish a diverse participant pool to the greatest extent possible. The study used partial disclosure because informing participants in advance that the task was designed to induce frustration could have altered their expectations and emotional responses, thereby reducing the validity of the experimental manipulation. The use of partial disclosure, the short duration of the frustration-induction procedure, the expected minimal-risk nature of the task, and the planned debriefing procedure were described in the ethics application and reviewed and approved by the Swedish Ethical Review Authority. Immediately after completing the task and all post-task questionnaires, participants were fully debriefed. The debriefing explained the study’s true purpose, the rationale for using partial disclosure, and the frustration-inducing elements of the FIT. Participants were given the opportunity to ask questions. The study procedure, including the use of partial disclosure and debriefing, was approved by the Swedish Ethical Review Authority.

Participants were informed that they would receive a gift card valued at either 50 or 100 SEK, depending on task performance. All participants received compensation, and the difference between compensation levels was kept small to avoid undue influence or coercion. The performance-contingent component was included as a mild experimental feature to increase task involvement, and goal obstruction may be accompanied by perceived gains or losses that affect the reward system. Table 1 displays a descriptive summary of participants’ personality traits and behavioral tendencies.

**Table 1:** Participants’ Descriptive Information.

Personality Traits	Mean	Median	SD
Extraversion	2.74	2.75	0.75
Agreeableness	3.36	3.39	0.67
Conscientiousness	2.78	2.75	0.74
Neuroticism	2.57	2.64	0.94
Open Mindedness	3.70	3.73	0.57
**Behavioral Tendencies**			
Internal Control	2.85	2.81	0.78
Perfectionism – Concern Over Mistakes	2.40	1.78	0.87
Perfectionism – Personal Standards	3.32	3.39	0.71
Emotional Intelligence	4.35	4.42	0.97
Discomfort Intolerance	2.29	2.25	0.62
Self-Esteem	1.63	1.59	0.53
Difficulties Engaging	2.91	3.00	0.87
Impulse Control Difficulties	1.95	1.18	0.78
BIS	1.71	1.60	0.50
BAS – Reward	1.64	1.56	0.47
BAS – Drive	2.44	2.38	0.72

**Note:** All values are presented on the original response scales of their respective questionnaires.

Personality traits were evaluated on a validated five-point response scale, BFI-2-XS [24], where participants showed the highest average scores for open-mindedness (*M* = 3.70, *SD* = 0.57) and agreeableness (*M* = 3.36, *SD* = 0.67). In contrast, neuroticism (*M* = 2.57, *SD* = 0.94), extraversion (*M* = 2.74, *SD* = 0.75), and conscientiousness (*M* = 2.78, *SD* = 0.74) showed moderate average levels. Behavioral tendencies such as internal control, perfectionism, emotional intelligence, and emotional regulation (impulse control difficulties and difficulties engaging) were evaluated on a validated five-point scale. Participants exhibited elevated emotional intelligence (*M* = 4.35, *SD* = 0.97) and personal standards for perfectionism (*M* = 3.32, *SD* = 0.71), with comparatively lower scores on impulse control difficulties (*M* = 1.95, *SD* = 0.78). Other behavioral traits, such as discomfort intolerance, self-esteem, and sensitivity to behavioral inhibition (BIS) and activation (BAS), were measured on four-point scales. Emotional challenges such as low self-esteem (*M* = 1.63, *SD* = 0.53) and moderate BIS sensitivity (*M* = 1.71, *SD* = 0.50) were observed.

### Procedures

The study was conducted in a controlled laboratory environment to ensure consistency across testing conditions. Upon arrival, participants were welcomed and provided an overview of the study’s general purpose as described in the consent form. After giving written informed consent, they completed a multi-stage experiment to assess frustration in response to a navigation-based novel Frustration Induction Task (FIT) [22].

The procedure was divided into three phases. First, in the pre-task assessment, participants completed a baseline questionnaire. Next, participants engaged in the FIT on an iPad. In the final post-task assessment, participants completed a questionnaire evaluating their emotional responses to the FIT, their overall frustration, and individual differences across personality traits and behavioral tendencies. Thus, the procedure followed a fixed sequence for all participants: consent and baseline assessment, completion of the FIT, post-task assessment of frustration and affective responses, individual-difference questionnaires, debriefing, and compensation.

### Measures

To assess individual readiness and baseline characteristics before frustration induction, participants completed a pre-task assessment that captured demographic information, current mood, physical energy, sleep quality, hunger levels, and familiarity with navigation tools and environments. The core of the experiment was a ten-trial iPad-based navigation task designed to induce frustration [22]. Using an ABC keyboard layout, participants were asked to enter various address names. The first two trials were free of manipulation, but the remaining eight trials incorporated layered components to gradually increase frustration. These frustration components included a 30-second time limit to induce time pressure, keyboard manipulations to produce case-sensitive errors, distracting visual elements such as running banners and warning banners, and a performance-based reward system that created a loss-framing element; participants earned compensation based on successful completion of each trial, making task failure more consequential. Following each trial, participants responded to a brief in-app questionnaire of five items rated on a 7-point Likert scale (1 = strongly disagree; 7 = strongly agree). They reported perceived difficulty (“I found the trial difficult to accomplish”), familiarity with the address, outcome alignment (“The trial result was as I expected”), frustration (“I found this trial frustrating”), and task motivation (“I am motivated to continue with the next trial”).

Frustration was operationalized as participants’ self-reported emotional response to the obstruction of goal-directed task performance during the FIT. Trial-level frustration was assessed after each trial using the item “I found this trial frustrating,” rated on a 7-point Likert scale from 1 = strongly disagree to 7 = strongly agree. Overall frustration was assessed after the task using a 7-point rating scale ranging from 1 (not frustrated) to 7 (very frustrated). These trial-level and overall ratings served as the primary indicators of participants’ frustration responses.

After the task, participants completed a comprehensive post-task questionnaire assessing emotional responses, behavioral tendencies, personality traits, frustration components, and overall frustration. An open-ended question at the beginning of the post-task questionnaire asked participants what they believed the study’s purpose was. This item was included as an awareness check to assess whether participants had guessed the task’s frustration-induction purpose before debriefing. These responses were not subjected to thematic or qualitative analysis and were not included in the primary statistical analyses. Then, to quantify their overall frustration levels, the participants first rated their experience on a 7-point Likert scale (1 = not frustrated, 7 = very frustrated). Participants also rated their perceived frustration on a 7-point scale from 1 (extremely negative) to 7 (extremely positive). To assess participants’ emotional responses associated with frustration during the task, post-task affective responses were assessed using the Positive and Negative Affect Schedule (PANAS) [25], rating the intensity of 20 affective states on a 5-point scale 1(very slightly or not at all) to 5 (very much). The scale included positive affect items, such as enthusiastic, determined, alert, attentive, and interested, and negative affect items, such as irritable, distressed, hostile, nervous, ashamed, guilty, scared, and afraid. The PANAS was included to characterize the affective profile accompanying frustration after the FIT, rather than to operationally define frustration itself.

To explore behavioral tendencies that may modulate frustration responses, participants completed several validated psychological scales. The internal control scale, Locus of Control [26], assessed beliefs about personal agency and outcome control, using six levels of agreement for items like “When I make plans, I am almost certain to make them work,” “My life is determined by my own actions,” etc. The Frost Multidimensional Perfectionism Scale [27] measured two perfectionism subdomains: concern over mistakes and personal standards, on a 5-point Likert scale from “strongly disagree” to “strongly agree.” Sample items included “If I fail at school, I am a failure as a person,” “I set higher goals than most people,” etc. To measure discomfort intolerance, the Frustration Discomfort Scale [16] was administered, where participants rated the strength of belief on a scale from 0 (absent) to 4 (very strong) for items such as “I can’t stand doing tasks that seem too difficult,” “I need the easiest way around problems.”

Emotion regulation was assessed using two subscales from the Difficulties in Emotion Regulation Scale [28], which evaluated impulse control difficulties and difficulties engaging in goal-directed behavior. Items such as “When I’m upset, I have difficulty concentrating,” “When I’m upset, I lose control over my behaviors,” etc., were rated on a 5-point scale, ranging from 1 (almost never) to 5 (almost always). Emotional intelligence was measured using the Emotional Skills and Competence Questionnaire [29], which assessed the ability to perceive, express, and regulate emotions. Responses ranged from 1 (strongly disagree) to 5 (strongly agree) for items like “I am able to express my emotions well,” “I can maintain a good mood even if something bad happens,” etc. Self-esteem was measured using the Rosenberg Self-Esteem Scale [30], a 10-item measure rated on a 4-point scale, including positively and negatively phrased statements (e.g., “I take a positive attitude toward myself”, “I wish I could have more respect for myself.”)

Motivational sensitivity was assessed using the Behavioral Inhibition System and Behavioral Activation System scales (BIS/BAS) [31]. The BIS scale assesses sensitivity to punishment, threat, and behavioral inhibition, whereas the BAS scales assess reward-related motivation, including reward responsiveness, drive, and fun seeking. In the present study, BIS/BAS scores were used as dimensional indicators of individual differences in motivational sensitivity rather than as diagnostic or psychiatric classifications. The BIS subscale focused on behavioral inhibition (e.g., “Criticism or scolding hurts me quite a bit”, “I have very few fears compared to my friends”). In contrast, the BAS subscales covered reward responsiveness, drive, and fun seeking (e.g., “I crave excitement and new sensations,” “I go out of my way to get things I want,” “I often act on the spur of the moment”), all rated on a 4-point Likert scale ranging from 1 (strong agreement) to 4 (strong disagreement).

Additionally, personality traits were assessed using the Big Five Inventory–2 Extra-Short Form (BFI-2-XS) [24], a brief self-report measure of five broad personality dimensions: extraversion, agreeableness, conscientiousness, neuroticism, and open-mindedness. Each trait was assessed dimensionally, with higher scores reflecting greater endorsement of the corresponding trait. The BFI-2-XS was selected because it provides a brief assessment of broad personality traits while minimizing participant burden in a multi-measure experimental protocol. BFI-2-XS includes a 15-item scale measuring five core personality traits (extraversion, agreeableness, conscientiousness, neuroticism, and open-mindedness). Each trait was assessed via three statements (e.g., “Is full of energy,” “Is reliable,” “Worries a lot”), rated from 1 (disagree strongly) to 5 (agree strongly).

They then evaluated how frustrating each task component was, such as time pressure, keyboard manipulations, visual interference (running banner and warning banner), or environmental distractions like noise or lighting, using the same 1–7 point Likert scale. To assess awareness of task manipulations, they rated their recognition of keyboard errors (“Did you notice the keyboard was not functioning correctly?”) and their confidence that the task had been intentionally manipulated (“How confident are you that the keyboard was manipulated during the task?”), both on a 7-point scale. After completing all measures, participants were fully debriefed and compensated for their task performance.

### Data analysis

To analyze frustration clusters in the dataset, we used a combination of software tools and statistical methods. All primary analyses were conducted in Python (version 3.13), using several key libraries for data processing, statistical modeling, and visualization. Pandas was used for data organization and manipulation, while NumPy handled numerical computations. Visualizations, including heatmaps, scatter plots, radar plots, and bar graphs, were created using the matplotlib and seaborn libraries. For clustering analyses, we used SciPy’s linkage and fcluster functions to perform hierarchical clustering with complete linkage and Euclidean distance as the similarity metric. To explore the relationship between questionnaire responses and frustration levels, we applied Partial Least Squares Regression using the PLS regression function from the Scikit-learn (Sklearn) library. Sklearn also supported various data preprocessing tasks throughout the analysis. Because participants were recruited using a non-probability convenience sampling strategy, inferential analyses were interpreted as exploratory rather than as population-representative estimates. In addition, because the number and size of the frustration profiles were empirically derived rather than prespecified, all cluster-based group comparisons were interpreted as exploratory follow-up analyses. Welch’s ANOVA and Games-Howell post hoc tests were used where appropriate because these methods are robust to unequal group sizes and heterogeneity of variance. False discovery rate correction was applied within test families to reduce the likelihood of false-positive findings across multiple comparisons.

The data processing began with cleaning and transforming the raw dataset. Since the original data was collected in spreadsheet format, initial adjustments were made in Microsoft Excel. This included reverse-scoring negatively worded items (for example, personality questions where higher scores indicated a greater degree of that trait) to ensure consistency in variable interpretation. Additional processing steps were performed after the data was imported into Python. Descriptive statistics (means and standard deviations) were reported on the original response scales of their respective questionnaires to facilitate interpretation. For multivariate analyses and graphical visualization, continuous variables were rescaled to a common 0–10 metric using min–max normalization to enable comparisons across measures originally recorded on different response scales. The transformation was applied as ${\text{X}}^{\prime }=\frac{\text{X }-\text{Xmin}}{\text{Xmax }-\text{ Xmin}}\;$, where X is the observed value, X_min_ and X_max_ are the theoretical minimum and maximum possible values for that measure, and X′ is the rescaled value. For example, Likert-type responses originally recorded on 1–5 or 1–7 scales were proportionally mapped to the 0–10 metric. This rescaling preserved the relative distribution of each variable while enabling direct comparability across measures in subsequent analyses.

Additional normalization steps were applied to the radar plots to ensure consistent scaling across variables. After the initial normalization, which scaled all values to 0–1, the values were multiplied by 10 to align with the radar plot axes, which ranged from 0 to 10. This procedure was applied to personality traits, behavioral tendencies, perceived task triggers, and emotional response variables to ensure comparability across measures originally recorded on different scales. Standard deviations for each variable were scaled similarly and plotted as dotted lines to illustrate the variability within each cluster; graphical conventions, including color coding and line styles, are specified in the corresponding figure’s legends. For hierarchical clustering, normalized frustration scores were used to generate dendrogram structures, from which three distinct clusters emerged. These were labeled as High, Mid, and Low frustration based on the ranked mean frustration scores of each cluster. To explore relationships between frustration and other factors, we conducted Pearson correlation coefficients and dimensionality reduction with PCA.

To evaluate whether the identified frustration clusters differed across demographic variables, personality traits, behavioral tendencies, perceived task triggers, and emotional responses, we conducted inferential statistical analyses in Python. Gender distribution across clusters was examined using a chi-square test of independence, with Cramér’s V reported as an effect size. Age differences across clusters were examined using Welch’s ANOVA (robust to unequal variances and unequal group sizes), reporting partial eta squared (ηp²).

For conceptually related outcome sets (Big Five traits; perceived frustration triggers; emotional responses; behavioral tendencies), we first conducted multivariate analyses of variance (MANOVAs) with cluster membership as the between-subjects factor and Wilks’ Lambda (Λ) as the primary multivariate test statistic. When the MANOVA indicated a significant multivariate cluster effect, follow-up Welch ANOVAs were performed for each variable within that outcome set. To control for multiple comparisons, false discovery rate correction (Benjamini–Hochberg) was applied within each family of tests (traits, triggers, emotions). For follow-up tests, partial eta squared (ηp²) was reported.

When Welch ANOVAs indicated statistically significant effects after false discovery rate correction, Games–Howell post-hoc comparisons were conducted to examine pairwise differences between clusters. For each comparison, mean differences with corresponding 95% confidence intervals (CIs) and exact p values were reported. Confidence intervals were computed based on the standard error of the mean difference. All inferential tests were two-tailed.

## Results

### Frustration clusters and demographics

To visualize the separation of the frustration clusters identified through hierarchical clustering, we conducted a principal component analysis (PCA) on the normalized frustration variables. PCA was used solely as a dimensionality-reduction technique for visualization and did not contribute to cluster identification.

The hierarchical clustering identified three frustration clusters: High Frustration (n = 96, 66.2%), Mid Frustration (n = 39, 26.9%), and Low Frustration (n = 10, 6.9%). The proportion of female participants was similar across profiles: 66.7% in the High Frustration profile, 71.8% in the Mid Frustration profile, and 80.0% in the Low Frustration profile, as presented in Table 2. Age did not differ substantially across clusters, with means ranging from 25.0 to 28.8 years. To examine whether demographic characteristics differed across frustration clusters, inferential analyses were conducted. A chi-square test of independence indicated no significant association between gender and frustration cluster, *χ²* (2) = 0.95, *p = .62*, Cramér’s V = .08. Similarly, a Welch’s ANOVA revealed no significant difference in age across clusters, *F* (2, 23.38) = 0.58, *p* = .57, partial η² = .01 (*N* = 145). Thus, profile membership was not significantly associated with gender, and no significant age differences were detected across profiles in this sample. Educational background showed modest variation: the Low Frustration cluster had the highest proportion of participants with advanced degrees (40%), compared with 30% in the High group and 28% in the Mid group.

**Table 2:** Demographic information by frustration clusters.

Variables	High Frustration	Mid Frustration	Low Frustration
Participants	96 (66.2%)	39 (26.9%)	10 (6.9%)
Men	32 (33.3%)	11 (28.2%)	2 (20.0%)
Women	64 (66.7%)	28 (71.8%)	8 (80.0%)
Age, M (SD)	28.52 (9.19)	28.82 (8.77)	25.00 (10.24)
Men Age, M (SD)	30.81 (11.48)	28.55 (5.57)	21.00 (0.00)
Women Age, M (SD)	27.35 (7.62)	28.93 (9.87)	26.00 (11.36)

**Fig 1:**
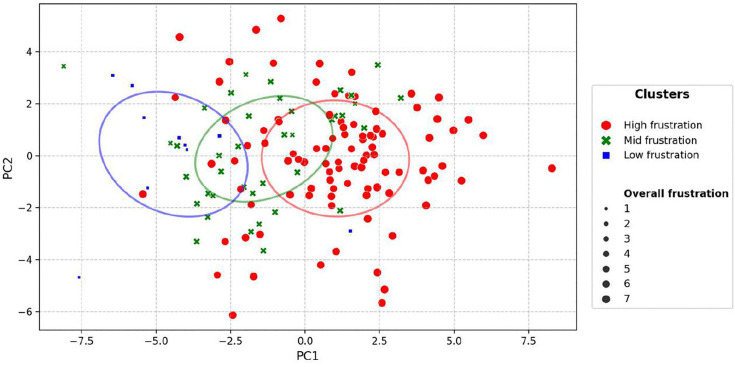
Principal Component Analysis of frustration clusters. (1a) Scatterplot of the first two principal components of normalized frustration scores, color-coded by cluster: High Frustration (red), Mid Frustration (green), and Low Frustration (blue). (1b) Each point represents a participant, with point size scaled to the overall frustration score (1–7). (1c) Ellipses indicate the covariance spread of each cluster.

Fig 1 displays participants’ scores on the first two principal components, color-coded by frustration profile. PC1 explained 32.5% of the variance, and PC2 explained 18.7%, together accounting for 51.2% of the total variance. Participants in the High Frustration group (red) clustered toward the positive end of PC1, while the Low Frustration group (blue) appeared more on the negative side. The Mid Frustration group (green) was distributed between the two. The High Frustration group exhibited the broadest variability, whereas the Low Frustration group showed more compact in the PCA visualization; however, this pattern should be interpreted cautiously because the profile contained only 10 participants.

### Personality traits across clusters

To examine whether personality traits differed across frustration profiles, we compared distributions of the Big Five traits (extraversion, agreeableness, conscientiousness, neuroticism, and openness) among the High, Mid, and Low Frustration clusters.

A MANOVA revealed a statistically significant multivariate effect of cluster on the combined Big Five personality traits, Wilks’ Λ = .88, *F* (10, 276) = 1.88, *p* = .047, partial η² = .06 (*N* = 145), indicating modest overall differences in trait profiles across frustration groups. However, follow-up Welch ANOVAs with false discovery rate (FDR) correction did not identify statistically significant differences for any individual trait (all *p* > .05; Table 3). Although conscientiousness and neuroticism showed nominal differences at the uncorrected level, these effects were not statistically significant after correction.

**Table 3:** Big Five Traits.

Measure	df1	df2	F	p before FDR	ηp²	Significant FDR	p after FDR
Conscientiousness	2	23.75	5.179	0.014	0.071	False	0.068
Neuroticism	2	24.92	4.044	0.030	0.050	False	0.075
Agreeableness	2	23.61	1.897	0.172	0.021	False	0.287
Extraversion	2	24.87	0.501	0.612	0.007	False	0.632
Openness	2	24.03	0.467	0.632	0.006	False	0.632

**Fig 2:**
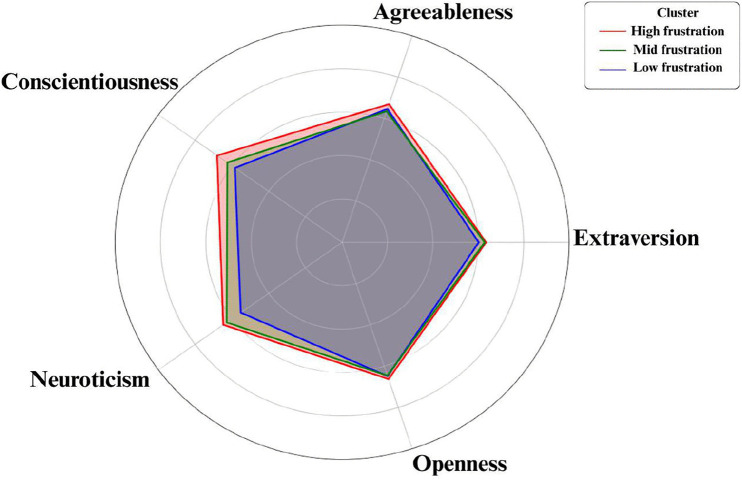
Personality traits across frustration clusters. (2a) Radar plot of normalized Big Five personality trait scores in High (red), Mid (green), and Low (blue) Frustration clusters. (2b) Neuroticism and conscientiousness showed the largest descriptive differences across clusters, whereas extraversion, agreeableness, and openness remained comparatively similar across profiles.

Descriptively, neuroticism displayed the largest separation between clusters in Fig 2. The High Frustration group reported higher neuroticism (*M* = 7.8, *SD* = 1.2) compared with the Mid (*M* = 6.5, *SD* = 1.0) and Low clusters (*M* = 5.2, *SD* = 0.9). Pairwise Games–Howell comparisons indicated that the High cluster differed from the Low cluster (mean difference = 0.957, 95% CI [0.300, 1.614], p = .037), whereas differences between High and Mid clusters were not statistically significant (p = .560). Conscientiousness showed a similar directional gradient (High: *M* = 8.0, *SD* = 1.1; Mid: *M* = 7.2, *SD* = 0.8; Low: *M* = 6.8, *SD* = 0.7). However, because no individual trait remained significant after FDR correction, these patterns should be interpreted as descriptive rather than inferential.

Extraversion, agreeableness, and openness were relatively stable across clusters, with small mean differences and confidence intervals spanning zero. Overall, personality differences across frustration profiles were subtle and of limited magnitude.

To assess how situational factors contributed to frustration, we examined participants’ ratings of task-related components in the FIT. The goal was to determine whether specific triggers, such as time pressure, keyboard manipulation, or address complexity, differed in the strength with which they elicited frustration across the High, Mid, and Low Frustration clusters.

We calculated mean and standard deviation values for each trigger. A MANOVA demonstrated a statistically significant multivariate effect of cluster on perceived task-related triggers, Wilks’ Λ = .61, *F* (20, 266) = 3.67, *p* < .001, partial η² = .22 (*N* = 145), indicating substantial differences in trigger sensitivity across frustration profiles. Follow-up Welch ANOVAs with FDR correction revealed statistically significant cluster effects for time pressure, final performance summary, warning banner, ABC keyboard layout, keyboard manipulation, failure point, and running banner (all adjusted *p* < .05; Table 4). No significant differences were observed for familiarity with the address, long address length, or reward system.

**Table 4:** Frustration Triggers.

Measure	df1	df2	F	p before FDR	ηp²	Significant FDR	p (FDR)
Time Pressure	2	25.19	15.703	0.000	0.154	True	<0.001
Final Performance Summary	2	24.12	16.411	0.000	0.184	True	<0.001
Warning Banner	2	22.18	13.901	0.000	0.196	True	<0.001
ABC Keyboard	2	23.17	9.838	0.001	0.130	True	0.002
Keyboard Manipulation	2	20.99	10.361	0.001	0.200	True	0.002
Failure Point	2	23.73	9.515	0.001	0.121	True	0.002
Running Banner	2	25.87	5.562	0.010	0.054	True	0.014
Reward System	2	24.73	2.212	0.131	0.027	False	0.163
Long Address	2	23.67	1.876	0.175	0.027	False	0.195
Familiarity Address	2	25.45	0.664	0.523	0.008	False	0.523

**Fig 3:**
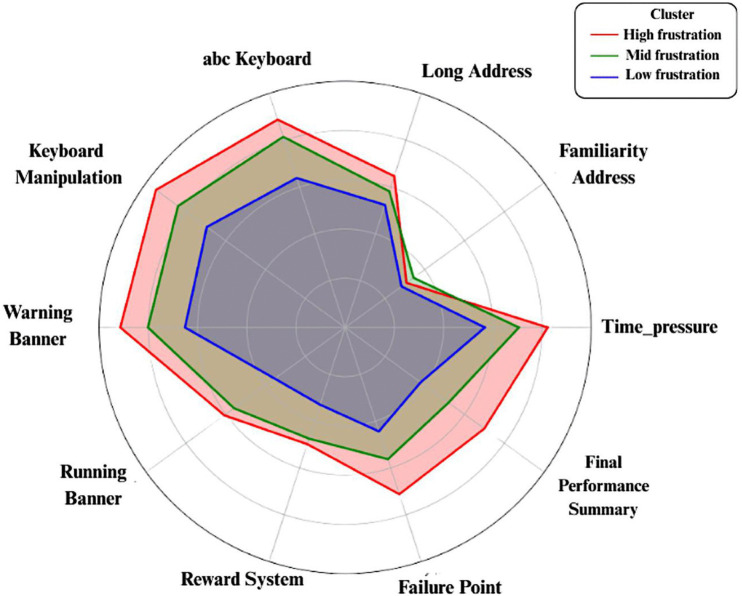
Frustration triggers across clusters. (3a) Radar plot of normalized ratings of FIT components, including address familiarity, address length, time pressure, keyboard manipulation, and reward system disruptions, for High (red), Mid (green), and Low (blue) Frustration clusters.

Games–Howell comparisons indicated that the High Frustration cluster consistently reported elevated trigger sensitivity relative to the Low cluster in Fig 3. For example, perceived time pressure was markedly higher in the High group than in the Low group (mean difference = 2.982, 95% CI [1.850, 4.113], *p* < .001) and also higher than in the Mid group (mean difference = 1.367, 95% CI [0.553, 2.181], *p* = .005). Similarly, frustration associated with the final performance summary was greater in the High cluster compared with the Low cluster (mean difference = 3.735, 95% CI [1.943, 5.527], *p* = .005) and the Mid cluster (mean difference = 2.100, 95% CI [1.253, 2.947], *p* < .001).

Keyboard-related disruptions followed the same pattern. The High cluster reported significantly greater frustration than the Low cluster for keyboard manipulation (mean difference = 2.987, 95% CI [1.277, 4.698], *p* = .018) and warning banners (mean difference = 3.068, 95% CI [1.739, 4.397], *p* = .003). In contrast, address familiarity ratings were statistically comparable across clusters, and all pairwise confidence intervals including zero (all *p* > .60). Descriptive means reflected in this stability (High: *M* = 4.5, *SD* = 1.0; Mid: *M* = 4.6, *SD* = 0.9; Low: *M* = 4.4, *SD* = 0.8). Overall, heightened frustration was characterized by amplified sensitivity to time constraints, performance evaluation, and interface disruptions, whereas familiarity-related task elements did not differentiate the groups.

### Behavioral tendencies across clusters

To investigate whether behavioral tendencies differentiated frustration responses, we compared scores for internal control, emotional intelligence, perfectionism, discomfort intolerance, self-esteem, impulse control difficulties, difficulties engaging, and BIS/BAS sensitivity across the three frustration clusters.

A MANOVA examining internal control, perfectionism dimensions, discomfort intolerance, emotional intelligence, self-esteem, impulsive control difficulties, engagement difficulties, and BIS/BAS indices did not yield a statistically significant multivariate cluster effect, Wilks’ Λ = .79, *F* (24, 262) = 1.37, *p* = .123, partial η² = .11 (*N* = 145). Consistent with the multivariate result, follow-up Welch ANOVAs with FDR correction indicated no statistically significant between-cluster differences for individual behavioral tendencies (all adjusted *p* > .05; Table 5).

**Table 5:** Behavioral Tendencies.

Measure	df1	df2	F	p before FDR	ηp²	Significant FDR	p (FDR)
Perfectionism (Concern Over Mistakes)	2	32.82	5.085	0.012	0.027	False	0.079
Impulse Control Difficulties	2	27.09	4.818	0.016	0.041	False	0.079
Difficulties Engaging	2	24.04	4.642	0.020	0.062	False	0.079
BIS (Behavioral Inhibition)	2	24.96	3.046	0.066	0.037	False	0.157
BAS Fun Seeking	2	24.54	3.066	0.065	0.037	False	0.157
Discomfort Intolerance	2	23.4	2.348	0.118	0.037	False	0.221
Emotional Intelligence	2	26.17	2.216	0.130	0.02	False	0.221
Perfectionism (Personal Standards)	2	24.79	1.728	0.198	0.02	False	0.275
BAS (Behavioral Activation)	2	26.25	1.676	0.207	0.021	False	0.275
Self-esteem	2	32.93	1.266	0.295	0.015	False	0.354
BAS Reward Responsiveness	2	24.67	0.741	0.487	0.01	False	0.531
Internal Control	2	22.37	0.289	0.752	0.005	False	0.752

**Fig 4:**
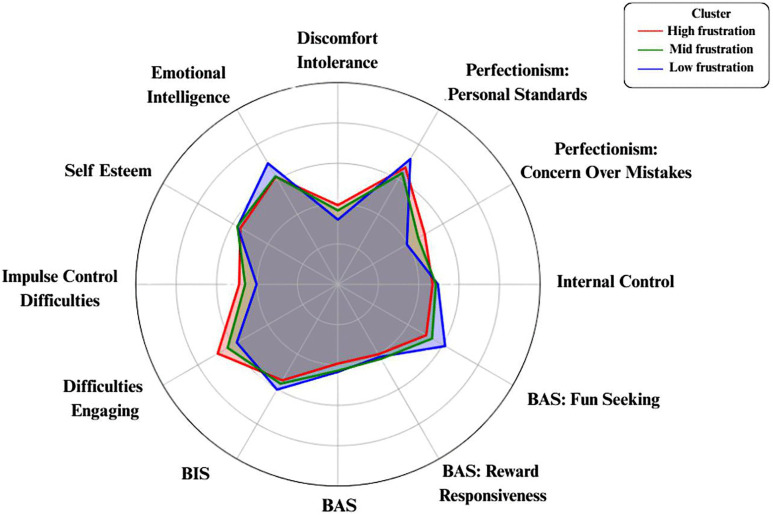
Behavioral tendencies across frustration clusters. (4a) Radar plots of internal control, emotional intelligence, perfectionism (personal standards and concern over mistakes), discomfort intolerance, self-esteem, impulse control difficulties, difficulties engaging, and BIS/BAS sensitivity in High (red), Mid (green), and Low (blue) Frustration clusters.

Descriptively, emotional intelligence appeared highest in the Low Frustration cluster (*M* = 8.5, *SD* = 0.8), followed by the Mid cluster (*M* = 7.3, *SD* = 1.0), and lowest in the High cluster (*M* = 6.2, *SD* = 1.2). Similarly, perfectionism of personal standards and BIS sensitivity showed directional gradients across clusters. However, pairwise comparisons revealed small to moderate mean differences with confidence intervals frequently spanning zero. For example, the normalized emotional-intelligence mean was highest in the Low Frustration profile and lowest in the High Frustration profile (mean difference = 0.862, 95% CI [0.312, 1.411], *p* = .022). However, as shown in Fig 4, However, neither emotional intelligence nor any other individual behavioral tendency remained statistically significant after FDR correction. Therefore, these trends should be interpreted descriptively.

### Emotional responses across frustration clusters

To evaluate how emotional reactions varied across frustration profiles, we examined positive and negative affective ratings in the High, Mid, and Low Frustration clusters. The question was whether certain emotions, such as attentiveness, interest, or distress, were more strongly associated with high frustration compared to lower frustration states.

A MANOVA revealed a statistically significant multivariate effect of cluster on emotional responses following the task, Wilks’ Λ = .52, *F* (40, 246) = 2.37, *p* < .001, partial η² = .28 (*N* = 145), indicating substantial affective differentiation across frustration profiles. Follow-up Welch ANOVAs with FDR correction identified significant between-cluster differences in multiple negative affective states, including guilt, fear, scared, upset, irritability, hostility, shame, distress, jitteriness, and nervousness (all *p* < .01; Table 6). Positive engagement-related emotions including enthusiasm, determination, interest, and attentiveness did not show statistically reliable differences after correction, although excited and alert demonstrated significant between-cluster variation (Table 6). Notably, levels of excitement and alertness were higher in the High Frustration cluster relative to the Mid cluster (both *p* < .05, FDR-corrected), suggesting that the profiles differed in arousal-related positive affect, although the specific pairwise differences require interpretation from the corresponding post hoc comparisons.

**Table 6:** Emotional Responses.

Measure	df1	df2	F	p before FDR	ηp²	Significant FDR	p (FDR)
Guilty	2	77.65	53.451	<0.001	0.097	True	<0.001
Scared	2	57.51	22.211	<0.001	0.051	True	<0.001
Afraid	2	69.69	28.46	<0.001	0.043	True	<0.001
Upset	2	23.42	21.734	<0.001	0.265	True	<0.001
Irritable	2	24.23	18.053	<0.001	0.202	True	<0.001
Hostile	2	31.19	12.705	<0.001	0.067	True	<0.001
Ashamed	2	28.48	12.252	<0.001	0.092	True	<0.001
Distressed	2	23.13	10.31	0.001	0.141	True	0.002
Jittery	2	24.11	7.824	0.002	0.099	True	0.005
Nervous	2	23.64	7.185	0.004	0.097	True	0.007
Excited	2	25.94	5.132	0.013	0.057	True	0.024
Alert	2	22.85	4.614	0.021	0.083	True	0.035
Strong	2	23.27	3.325	0.054	0.043	False	0.083
Proud	2	29.6	2.847	0.074	0.032	False	0.106
Active	2	24.83	2.191	0.133	0.027	False	0.177
Inspired	2	24.0	2.116	0.143	0.026	False	0.178
Enthusiastic	2	24.04	1.668	0.210	0.02	False	0.247
Interested	2	25.11	1.341	0.280	0.014	False	0.311
Determined	2	24.07	1.026	0.373	0.015	False	0.393
Attentive	2	23.28	0.394	0.679	0.007	False	0.679

**Fig 5:**
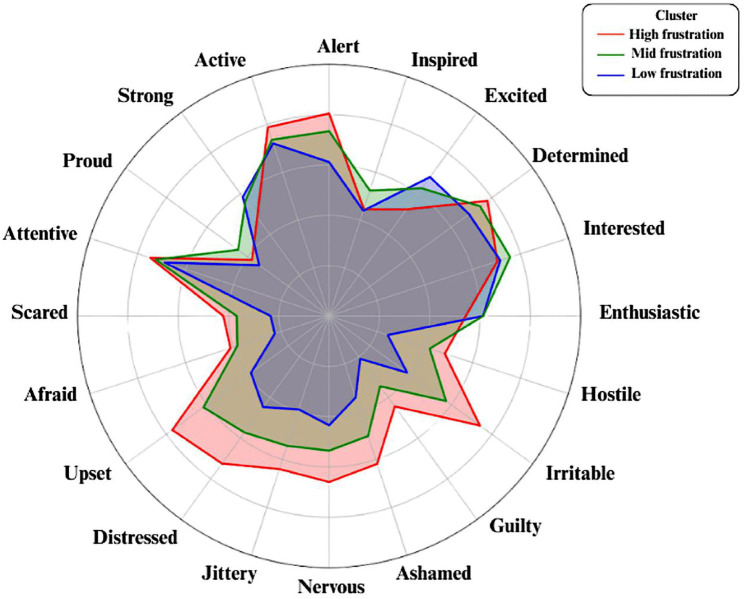
Emotional responses across frustration clusters. (5a) Radar plots of normalized ratings (0–10 scale) of positive and negative emotions, including attentiveness, interest, distress, irritability, guilt, nervousness, and enthusiasm, for High (red), Mid (green), and Low (blue) Frustration clusters.

Games–Howell comparisons indicated that negative emotions were consistently elevated in the High Frustration cluster relative to the Low cluster. For example, guilt was higher in the High cluster compared with the Low cluster (mean difference = 2.927, 95% CI [2.322, 3.533], *p* < .001) and compared with the Mid cluster (mean difference = 1.242, 95% CI [0.363, 2.121], *p* = .018). Similarly, irritability was markedly elevated in the High cluster relative to the Low cluster (mean difference = 4.477, 95% CI [2.741, 6.213], *p* < .001). Distress showed a comparable pattern, with the High cluster exceeding the Mid cluster (mean difference = 1.908, 95% CI [0.944, 2.872], *p* < .001) and the Low cluster (mean difference = 3.471, 95% CI [1.222, 5.721], *p* = .031). In contrast, attentiveness remained stable across groups (High: *M* = 8.0, *SD* = 0.7; Mid: *M* = 8.1, *SD* = 0.6; Low: *M* = 8.0, *SD* = 0.5), with no statistically significant pairwise differences. Overall, as shown in Fig 5, higher frustration was associated with increased negative affect and elevated arousal-related responses, while attentional engagement remained stable across clusters.

## Discussion

This study explored the psychological framework of frustration by examining whether broad personality traits, behavioral tendencies, and situational triggers are associated with distinct frustration responses. Using the FIT [22] in a controlled setting, participants were clustered based on their self-reported frustration levels, allowing us to compare dispositional characteristics, task-trigger sensitivity, and post-task affective responses across frustration profiles. The results offer new insights into the complex nature of frustration, revealing both expected and unexpected patterns, with the clearest differentiation emerging in task-trigger sensitivity and negative affective responses, whereas dispositional measures showed more modest or descriptive variation.

Identifying three distinct frustration clusters (High, Mid, and Low) supported the notion that frustration is not a uniform experience but varies along a spectrum influenced by individual differences in emotional sensitivity, cognitive control, and contextual cues. It also aligns with the contemporary frustration-aggression theory, which emphasizes how personality factors and the perceived significance of blocked goals impact emotional outcomes, with dispositional factors potentially shaping how these constraints are experienced [18]. The Low Frustration cluster contained a higher proportion of participants with advanced degrees; however, because education was examined descriptively, this pattern should be interpreted with caution. One possibility is that formal education is associated with improved executive functioning and metacognitive regulation (including planning, error monitoring, and inhibitory control), that which are essential for emotion regulation under cognitive strain [32], which could hypothetically contribute to lower frustration in demanding tasks. Age and gender did not differ across clusters, and inferential tests indicated no association between cluster membership and either variable. This suggests that these demographic factors alone do not sufficiently explain the observed variability in frustration, pointing toward more nuanced psychological profiles for understanding emotional regulation. This variability complicates intervention, as it suggests that individuals with high frustration are accompanied by elevations in multiple negative emotions, including “internalizers” (prone to inward states like guilt and shame) and “externalizers” (prone to outward states like irritability or aggression). Although the present analyses do not partition the High Frustration cluster into internalizing versus externalizing subgroups, the emotional pattern is consistent with heterogeneity in frustration expression described in broader psychopathology frameworks [33].

As predicted, personality traits emerged as modest differentiators across clusters at the multivariate level, but no individual Big Five trait remained statistically reliable after correction for multiple comparisons. Neuroticism showed the largest descriptive separation across clusters, with elevated mean scores in the high frustration cluster, echoing decades of literature linking neuroticism to heightened sensitivity to negative stimuli, and poor coping under stress [34], although it did not remain statistically reliable after multiple-comparisons correction. This trait has been associated with predisposing individuals to interpret ambiguous or adverse events more negatively, amplifying perceived threats or failures [15], which could plausibly contribute to stronger frustration responses when task conditions feel obstructive or evaluative and may explain their intensified frustration responses during the FIT task. Interestingly, conscientiousness showed a nominal directional gradient across clusters, which was an unexpected result. Conscientious individuals are typically characterized by persistence, organization, and goal-directed behavior [35], traits generally associated with higher frustration tolerance. However, one interpretation is that their strong goal orientation may increase frustration when performance is obstructed, especially under external constraints that differentiated clusters in this study (e.g., time pressure, evaluative feedback system manipulations). This aligns with the Goal Shielding Theory [36], which posits that individuals highly committed to task completion may become more emotionally reactive when obstacles threaten their goals. Thus, while conscientiousness is generally adaptive, in rigidly structured tasks it may lead to increased emotional strain when goals are blocked, though this interpretation remains tentative given the modest trait effects observed here. Extraversion, openness, and agreeableness remained relatively stable across clusters. While extraversion has been associated with higher positive affect and resilience [37], it may play a limited role in acute frustration contexts where external stimuli or proximal task constraints are more immediate determinants of affect [38]. Similarly, openness and agreeableness may contribute more to long-term stress recovery than to real-time moment-to-moment emotional modulation [39].

The analysis of situational frustration triggers yielded findings that were consistent with and expand prior models of technology-based frustration [3,4]. Cluster differences were most pronounced for time pressure, evaluative feedback (final performance summary), and interface disruptions (warning and running banners, keyboard layout and manipulation, and failure points), with the High Frustration group consistently reporting elevated sensitivity to these features. This aligns with Cognitive Load Theory [40], which suggests that increased cognitive demands and reduced perceived controllability can amplify negative affective responses. More broadly, task conditions that increased urgency and disrupted the execution of fluent actions may have heightened perceived goal blockage, thereby producing stronger emotional spillover in the High Frustration cluster. Frustration related to time pressure and visual interference (e.g., running banners and warning banners) differed significantly across clusters, with the High Frustration group reporting a reliably higher sensitivity. Time pressure, in particular, is well-established as a stressor that impairs executive functioning and emotion regulation [23]. In contrast, reward-system ratings did not differentiate across clusters, suggesting that evaluative feedback and control-disrupting task features were more central in distinguishing frustration profiles than reward-related elements per se [41]. Address familiarity did not differ across clusters, indicating that familiarity with task content did not buffer frustration under the broader manipulated task environment. This pattern is consistent with the Learned Helplessness Framework [42], which proposes that when individuals repeatedly perceive uncontrollable events, their prior competence or familiarity loses its regulatory effect.

Behavioral tendency measures showed descriptive variation across clusters, but the multivariate test was not significant, and no individual tendency remained statistically reliable after correction for multiple comparisons. Emotional Intelligence scores were descriptively higher in the Low Frustration group, a pattern consistent in direction with the proposed role of emotional intelligence as a resilience factor [43], although the present study did not provide statistically reliable evidence of a between-profile difference. EI enables individuals to accurately perceive, understand, and regulate their emotions and those of others, thus reducing the likelihood of escalating emotional responses in challenging situations [44]. Therefore, individuals with higher EI may manage expectations and reappraise setbacks more adaptively during challenging tasks, which could contribute to lower frustration responses. Discomfort intolerance and impulse control difficulties showed nominal between-cluster differences, consistent with prior literature linking these traits to low distress tolerance and emotional dysregulation [16,28]. Discomfort intolerance specifically reflects rigid thinking styles and the inability to tolerate ambiguity or task difficulty, which were core to the FIT’s manipulations [22]. However, these tendencies did not reliably differentiate clusters after correction. Although not statistically reliable in the present sample, it is possible that discomfort intolerance and impulse-control difficulties can plausibly intensify frustration as individuals become increasingly focused on escaping or avoiding discomfort rather than solving the task [45].

Counterintuitively, perfectionism, especially personal standards, showed descriptive variation across clusters. While perfectionism is often viewed as maladaptive [27], this aligns with a growing body of research distinguishing between adaptive and maladaptive perfectionism [46]. Conceptually, individuals with high personal standards but low concern for mistakes may view setbacks as performance feedback rather than failure, potentially enabling them to remain composed under pressure. This nuance underscores the importance of breaking down perfectionism into subcomponents when evaluating its emotional consequences, rather than treating perfectionism as a unitary maladaptive construct. BIS/BAS indices, including reward responsiveness, did not reliably differentiate clusters after correction, suggesting that reward sensitivity as measured here may not be a primary driver of profile separation, despite the classic Reinforcement Sensitivity Theory, which proposes that individuals high in reward sensitivity become more emotionally reactive when reward expectations are violated [31,47]. Instead, in our task, the most consistent differentiators were triggers, including time pressure, evaluative feedback, and interface disruptions, which may have amplified perceived goal blockage and negative affect in High Frustration participants.

Post-task emotional responses revealed distinct affective landscapes for each cluster. The High Frustration group reported elevated negative affective responses, including distress, irritability, hostility, nervousness, guilt, shame, upset, and jitteriness, emotions closely tied to low frustration tolerance and emotional dysregulation [34]. Engagement-related positive states (including interest, determination, enthusiasm, and attentiveness) did not differ reliably after correction, indicating that cluster differences reflected heightened negative affect rather than reduced engagement. Notably, attentiveness was uniformly high across groups. This potentially suggests that the frustration manipulation did not diminish task engagement, most likely due to the novelty of the app interface. High attentiveness in the High Frustration group may reflect cognitive fixation or perseverative control efforts, where frustrated individuals hyper-focus on task failure to regain control, which can maintain negative affect under goal blockage [5]. Finally, the elevation of shame and guilt in the High Frustration group highlights internalized affective responses that may have longer-term consequences [33]. In addition, excited and alert also differed across clusters, suggesting that heightened frustration was accompanied by elevated arousal rather than a uniform reduction in positive activation [48]. Their presence following a short-term task failure suggests that specific individuals may exhibit heightened affective reactivity to goal blockage and evaluation, which may represent a pathway that converts momentary frustration into deeper emotional distress.

This study advances an integrated understanding of frustration by showing that frustration profiles are most clearly differentiated by task-trigger sensitivity and negative affective reactivity, while broad personality traits and behavioral tendencies showed more modest or descriptive variation. Identifying frustration profiles characterized by heightened sensitivity to time pressure, evaluative feedback, and interface disruptions, alongside elevated negative affect, offers a path toward more tailored interventions across clinical, educational, and workplace settings. Future research and interventions aimed at reducing frustration-related impairments should consider these multilayered mechanisms. Emotion regulation training, cognitive reframing strategies, and personalized resilience programs may be most effective when calibrated to the severity of frustration, patterns of negative affective reactivity, and the specific situational triggers that most reliably amplify frustration.

### Limitations

While this study offers new insights into the psychological mechanisms behind frustration, several limitations warrant mention. First, relying on self-reported measures leaves room for biases, such as participants misjudging their own emotions or trying to present themselves in a better light, even though validated scales were used. Second, the sample size was modest and drawn from a narrow regional pool, which could limit the generalizability of these findings to other populations. Cluster sizes were also imbalanced, particularly for the Low Frustration group (n = 10), which may reduce power for detecting smaller between-cluster effects. Additionally, the large number of outcomes required correction for multiple comparisons; while this reduces false positives, it may obscure smaller effects that warrant replication with preregistered hypotheses. Third, although age did not significantly differ across frustration clusters in the present analyses, participants ranged from adolescence to older adulthood, and developmental differences in cognitive control, emotion regulation, stress physiology, and frustration tolerance may not be fully captured by the current design. Participants at the lower and upper ends of the age range may represent extreme values relative to the sample’s central tendency, and future studies should examine frustration profiles using age-stratified sampling, age-matched groups, or separate developmental cohorts. Fourth, although the self-report instruments used in this study are widely used in psychological research, the broad age range means their psychometric properties may not be identical across adolescents, adults, and older adults. These measures were used as dimensional individual-difference indicators rather than diagnostic or psychiatric tools. Future studies should examine measurement invariance across age groups or use age-specific validated instruments when comparing developmental cohorts. Fifth, the FIT allowed standardized exposure to the same frustration-inducing elements across participants; laboratory-based frustration may differ from real-world frustration. Future studies should examine whether the identified frustration profiles generalize to naturalistic settings. Finally, while the Frustration Task successfully provoked emotional reactions in a controlled setting, it can not fully capture the complexity of frustration as it plays out in real life, where challenges are often longer, less predictable, and shaped by social interactions. Future work should aim to recreate these dynamics in more naturalistic environments to better understand how frustration unfolds outside the lab.
